# Photoelectrochemical study of carbon-modified p-type Cu_2_O nanoneedles and n-type TiO_2−*x*_ nanorods for Z-scheme solar water splitting in a tandem cell configuration†

**DOI:** 10.1039/c8ra09403a

**Published:** 2019-05-02

**Authors:** Nelly Kaneza, Pravin S. Shinde, Yanxiao Ma, Shanlin Pan

**Affiliations:** Department of Chemistry and Biochemistry, The University of Alabama Tuscaloosa AL 35487-0336 USA span1@ua.edu

## Abstract

Nanostructured photoelectrodes with a high surface area and tunable optical and electrical properties can potentially benefit a photoelectrochemical (PEC) water splitting system. The PEC performance of a nanostructured photoelectrode is usually quantified in a standard three-electrode configuration under potential-assisted conditions because of the additional overpotentials for the two half-reactions of water splitting. However, it is a necessity to fully recognize their potential to split water under unassisted conditions by designing a tandem cell that can provide sufficient voltage to split water. Herein, we present a tandem cell consisting of carbon-modified cuprous oxide (C/Cu_2_O) nanoneedles and oxygen-deficient titanium dioxide (TiO_2−*x*_) nanorods for unassisted solar water splitting. The synthesized photoelectrodes were characterized by X-ray diffraction (XRD), scanning electron microscopy (SEM), transmission electron microscopy (TEM), Raman spectroscopy, and electrochemical impedance spectroscopy (EIS) techniques. The tandem cell performance was analyzed by measuring the current–voltage responses in various photoelectrode configurations to validate the collective contributions of both photoelectrodes to unassisted solar water splitting. The PEC properties of C/Cu_2_O nanoneedles coupled with TiO_2−*x*_ nanorods in a tandem configuration exhibited a photocurrent density of 64.7 μA cm^−2^ in the absence of any redox mediator and external bias. This photocurrent density can be further enhanced with an application of external bias. Moreover, the heterojunction formed by the above-mentioned nanostructured photoelectrodes in intimate contact and in the absence of water exhibited 2 μA cm^−2^ UV photoresponsivity at 1.5 V with promising rectifying characteristics of a diode.

## Abstract 2

Nanostructured photoelectrodes with high surface-area and tunable optical-electrical properties can potentially benefit a Z-scheme photoelectrochemical water splitting systems to generate solar fuels at no external bias.

## Graphical abstract

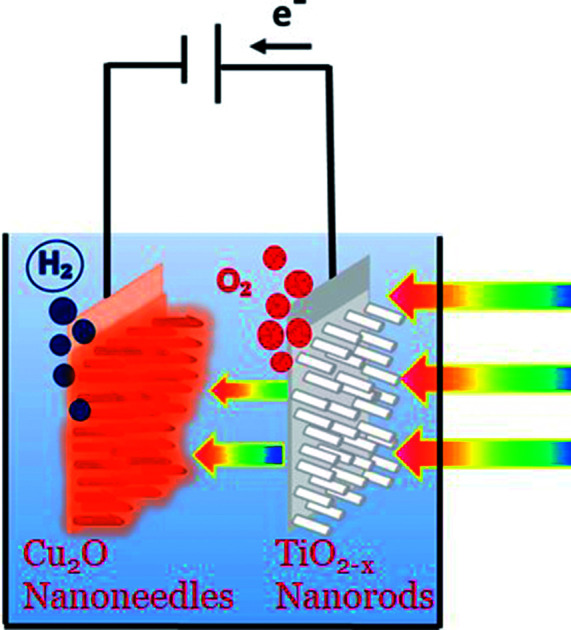

## 1. Introduction

With the ever-increasing global energy demands and the negative environmental impacts of fossil fuels, renewable solar energy has been regarded as an alternative energy source because of its abundance and wide availability.^1,2^ Among many techniques^3–5^ used to harvest and store solar energy, photoelectrochemical (PEC) water splitting offers an easy, low-cost and effective route to simultaneously produce hydrogen (H_2_), a clean fuel energy source, and oxygen (O_2_). First introduced in 1968,^6^ then popularized in 1972 by Fujishima and Honda,^7^ semiconductor-based solar water splitting has undergone considerable research and development.^8^ A typical PEC water splitting reaction, an uphill reaction, is shown as:1
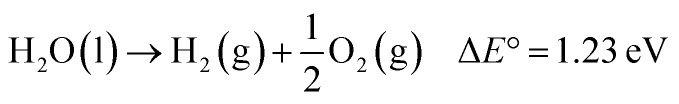


This process involves four fundamental steps: (1) light absorption by a semiconductor photoelectrode to generate electron–hole (e^−^/h^+^) pairs; (2) separation of the electron–hole pairs without recombination, the electrons are excited to the conduction band (CB) leaving the holes into the valence band (VB); (3) charge transport to respective electrode interfaces and surfaces; (4) water oxidation and reduction reactions by the separated charges on the semiconductor surface.^9–11^ Ideal semiconductor should have a band gap energy (*E*_g_) greater than 1.23 eV, a CB more negative than the reduction potential of H^+^/H_2_ (0 V *vs.* NHE), and a valence band more positive than the oxidation potential of O_2_/H_2_O (1.23 V *vs.* NHE).^11^

**Fig. 1:**
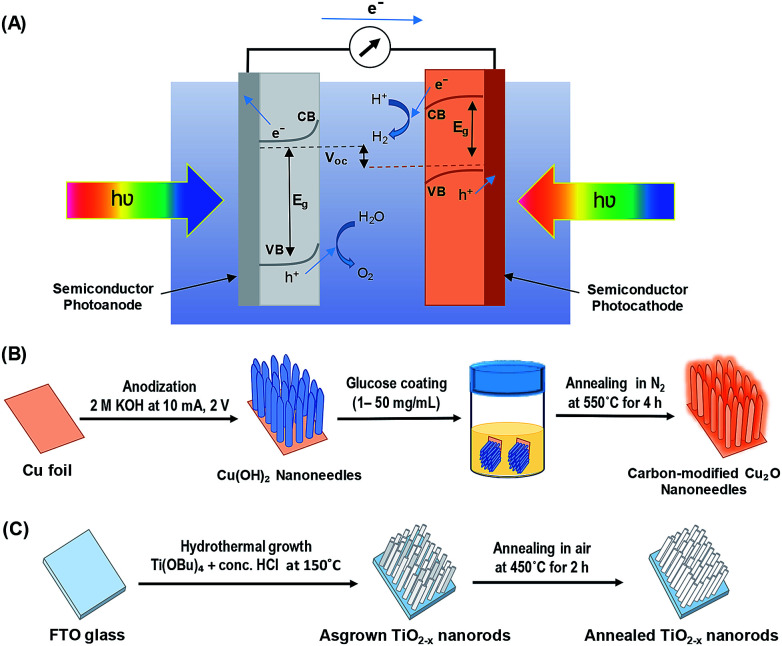
(A) Schematic of a self-biased Z-scheme system utilizing a photoanode and a photocathode under visible light illumination, (B) schematic illustration of carbon-modified Cu_2_O nanoneedles (NNs) synthesis, and (C) schematic illustration of hydrothermally grown oxygen-deficient TiO_2−*x*_ nanorods (NRs).

Great progress has been made toward the development of efficient and stable PEC systems, yet their performances are still very low because of the limited light absorption, charge separation, charge transport, sluggish kinetics of water splitting, and poor stability.^8,9,12^ In addition to requirements such as earth-abundance, stability, and low-cost; several approaches have been employed to develop active photocatalysts that can absorb solar energy in the entire UV-visible spectral region by including the use of heterojunction systems and nanostructure techniques.^10,13–16^ Inspired by nature's photosynthesis, a Z-scheme solar water splitting system can be established that involves two different photoactive semiconductors, a p- and n-type material, *via* a two-step excitation mechanism to enhance charge carrier-separation and hence improve the overall photoconversion efficiency.^13,15,17^Fig. 1A shows the schematic of a self-biased Z-scheme solar water splitting system involving p- and n-type photoelectrodes. In this approach, electron–hole pairs are generated in both the p- and n-type semiconductors, which absorb in different regions of the solar spectrum. Due to the band-bending at the semiconductor/electrolyte interface, the photogenerated holes react with water molecules generating oxygen (O_2_) molecules, while the photogenerated electrons in the photocathode move to the surface and reduce H^+^ to generate H_2_.^18^

For a successful self-biased Z-scheme PEC water splitting system, it is prerequisite that the Fermi energy (*E*_f_) level of the photoanode is at the higher energy level (more positive) than that of the photocathode.^18^ Our group recently reported unbiased Z-scheme solar water splitting system by combining an optimized Co-doped BiVO_4_/WO_3_ photoanode and CuO/CuBi_2_O_4_ photocathode in a two-electrode configuration; wherein both the photoelectrodes were illuminated with visible light.^19^ Such a two-electrode configuration holds a promise for providing enough voltage to split water in the absence of external bias. Titanium oxide (TiO_2_) and cuprous oxide (Cu_2_O) are another such photoanode,^20^ and photocathode^21^ materials that can potentially be used for Z-scheme solar water splitting as their energy band positions are sufficient enough to form a type II band alignment.^22^ Both the materials are earth-abundant, environmentally benign and low-cost. Cu_2_O (*E*_g_ = 2–2.2 eV) is an attractive p-type semiconductor, whose CB is appreciably negative (more energetic) than that of commonly employed visible-light semiconductors and the hydrogen evolution potential. Although it suffers from photo-corrosion,^24^ its surface can be protected by some means to utilize its full potential. The single or multi-layered protective materials such as TiO_2_,^23,24^ MoS_2_,^25,26^ carbon,^27,28^ graphene,^29–31^ molecular catalyst^32^ have been employed to protect Cu_2_O from photo-corrosion for solar water splitting and CO_2_ reduction reactions. TiO_2_ (*E*_g_ = 3.0–3.2 eV) is one of the best chemically stable materials as of yet with extensive applications. However, it has poor solar light absorptivity in the visible and NIR region.^33^ Although efforts were made to reduce its band gap to make it available in the visible region, TiO_2_ performs best as a UV-absorbing material and delivers relatively good photocurrents even at a small portion of UV light. Additionally, being a wide bandgap material, it can be used as a window layer to direct visible light on to the visible-light-absorbing material such as Cu_2_O in tandem configuration.

Herein, we report the PEC performance of carbon-modified p-type Cu_2_O nanoneedles (C/Cu_2_O NNs) and n-type oxygen-deficient TiO_2−*x*_ nanorods (TiO_2−*x*_ NRs) for unassisted Z-scheme solar water splitting system in a tandem configuration to efficiently utilize the sunlight absorption and improve the charge separation efficiency. A modification of Cu_2_O NNs with a thin protective carbon layer enhanced its photostability. A solution-based approach has been adapted to modify the Cu_2_O NNs to obtain corrosion-resistant carbon layer with enhanced conductivity for hydrogen evolution reaction.^27,28^ On the other hand, the introduction of oxygen vacancies in semiconductors such as TiO_2_ has proven to increase the electrical conductivity as well as charge transfer properties.^34,35^ In addition to this, the one-dimensional (1D) nanoscale morphology of both photocatalysts is employed to increase the surface area for multiplying scattering events to maximize the light absorption, promote the separation and migration of photogenerated charges, thereby accelerating the reaction rates to increase the photocurrent density.^14,23,36,37^

The efficiency of this tandem system is evaluated by placing the two photoelectrodes side by side (parallel) or one in the front of the other (tandem). On the other hand, since p–n heterojunctions have also received great interests due to their potential use in optoelectronic devices such as biosensors and photodiodes;^38–40^ in the present work, we also analyzed the electrical characteristics of a p-C/Cu_2_O NNs‖n-TiO_2−*x*_ NRs heterojunction photodiode behavior to further understand the charge transfer mechanism in the absence of water.

## 2. Experimental procedure

### 2.1 Synthesis of p-type C/Cu_2_O nanoneedles

All chemicals were purchased from Sigma-Aldrich and used directly without any treatment. The copper (Cu) sheets (99.9%, 0.0254 mm thick) were purchased from Alfa Aesar Co. Cu foils (1.5 cm × 1.5 cm) were first ultrasonically degreased in acetone, detergent water, and deionized water, respectively. The cleaned Cu foils were dried with nitrogen (N_2_) then electropolished in a solution mixture containing 55% H_3_PO_4_ and ethylene glycol (1.11 g mL^−1^).^41^ Then, the electropolished Cu foils were anodized in 2.0 M potassium hydroxide (KOH) aqueous solution at a current density of 10 mA cm^−2^ and a potential of 2.0 V at room temperature for 1–9 min.^42^ A light blue film, indicating the formation of Cu(OH)_2_ NNs, was formed on the surface of electropolished Cu foil; the anodized Cu foil was then rinsed with DI water and dried under N_2_ stream. The Cu(OH)_2_ NNs were then soaked in a dextrose solution (1–50 mg mL^−1^) for overnight, dried under N_2_ stream, then annealed at 550 °C for 4 h in N_2_ atmosphere to obtain yellowish-orange colored carbon (C)-modified Cu_2_O NNs.^28^Fig. 1B illustrates the schematic design of the formation of C/Cu_2_O NNs. Amount of carbon modification was varied by the concentrations of dextrose solution at 1, 3, 5, 7, 10, 15, 20 and 50 mg mL^−1^ to optimize the photocurrent and electrode stability. C_10_/Cu_2_O represents C/Cu_2_O NNs synthesized with 10 mg mL^−1^ dextrose with optimum activity in our following discussions.

### 2.2 Synthesis of n-type TiO_2−*x*_ nanorods array

TiO_2−*x*_ NRs array photoanodes were fabricated using a hydrothermal synthesis route.^43^ In a typical process (Fig. 1C), a mixture of 12 mL DI water and 12 mL concentrated hydrochloric acid (36.0–38.0 wt%) was poured into a Teflon-lined stainless steel autoclave of 50 mL capacity. The mixture was stirred for 5 min under ambient conditions, then 0.4 mL of titanium(iv) butoxide (Ti(OBu)_4_, 97.0%) was added to it and stirred for another 5 min. A cleaned piece (1.5 cm × 1.5 cm) of fluorine-doped tin oxide (FTO) substrate was then placed at an angle against the wall of the Teflon liner with conducting side facing down. The hydrothermal reaction was conducted for different durations (6–20 h) in an electric oven maintained at 150 °C. After the hydrothermal reaction, the autoclave was cooled to room temperature in the air; then the samples were rinsed with DI water and dried under N_2_ stream. As-synthesized TiO_2−*x*_ NRs were then annealed in air at 450 °C for 2 h at the ramp rate of 2 °C min^−1^.^44^

### 2.3 Sample characterization

The structure and morphology of the synthesized photoelectrodes were characterized by X-ray diffraction (XRD, Bruker D8 Discover with GADDS), scanning electron microscope (SEM, JEOL 7000 FE), transmission electron microscope (TEM, FEI Tecnai F-20), and Raman spectroscopy (Horiba Jobin Yvon LabRam HR800) using a 532 nm laser. The catalytic performances were tested electrochemically using cyclic voltammetry (CV) and linear sweep voltammetry (LSV) on a CHI760C electrochemical work station in a three or two-electrode cell configuration at room temperature. In a three-electrode configuration, C/Cu_2_O NNs or TiO_2−*x*_ NRs were used as the working electrode, a graphite rod as the counter electrode, and an Ag/AgCl (saturated KCl) as the reference electrode. An aqueous solution made of 0.5 M sodium sulfate (Na_2_SO_4_) and 0.1 M potassium phosphate monobasic (KH_2_PO_4_) was used as the electrolyte solution (pH 5.0) and was deaerated by purging N_2_ gas for at least 30 min before all the electrochemical and photoelectrochemical measurements. Electrochemical impedance spectroscopy (EIS) study of the samples was carried out using a three-electrode system on a CHI760C electrochemical work station in the frequency range of 100 kHz to 0.1 Hz with an AC potential amplitude of 10 mV and a DC potential of −0.3 V *vs.* Ag/AgCl for C/Cu_2_O samples and 0.6 V *vs.* Ag/AgCl for TiO_2−*x*_ samples. The Mott–Schottky (MS) analyses of C/Cu_2_O and TiO_2−*x*_ electrodes were performed at the 1 kHz frequency in their respective potential regions. The EIS and MS measurements were performed in buffered near-neutral 0.5 M Na_2_SO_4_ electrolyte. The optical properties of C/Cu_2_O NNs and TiO_2−*x*_ NRs samples were measured using UV-vis spectrophotometer (Varian Cary 50) and spectrofluorometer (Jobin Yvon Horiba fluoromax-3). The current–voltage (*J*–*V*) characteristics were measured using a Keithley 2400 source meter with the assistant of a LabVIEW program. A simulated solar light of the intensity of 100 mW cm^−2^ intensity generated from a solar simulator (Newport 66902, Xenon Arc lamp-modified with an Oriel 1.5 air mass, AM spectral filter).

## 3. Results and discussion

**Fig. 2:**
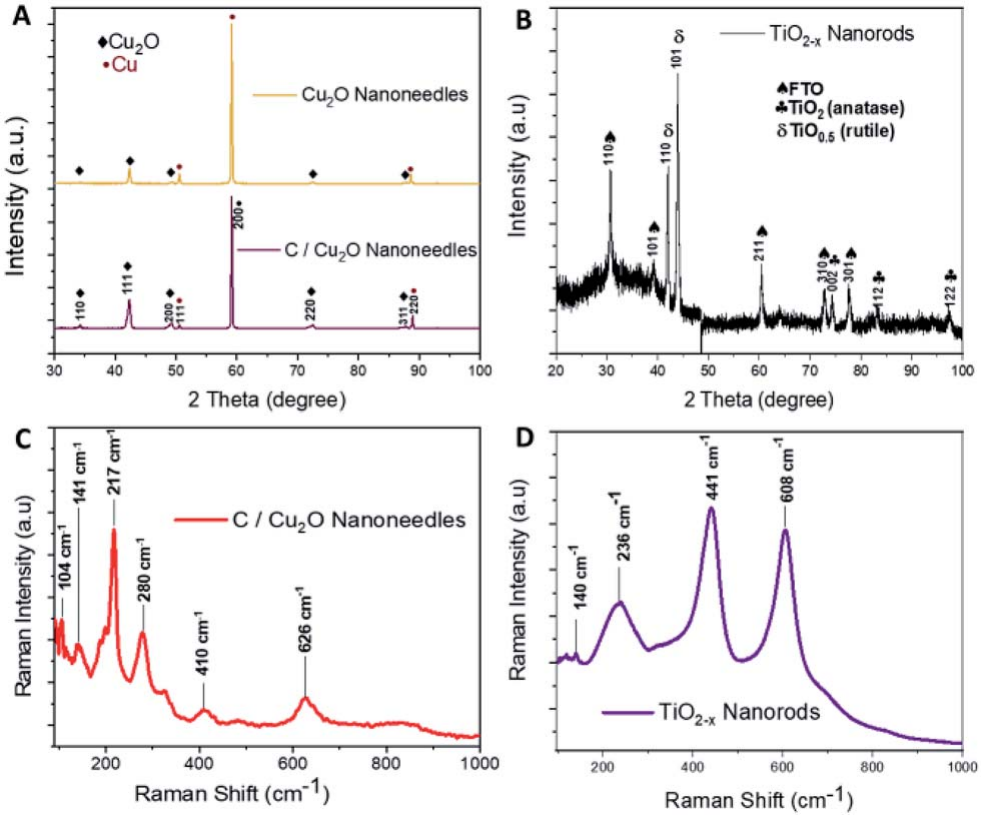
X-ray diffraction patterns of (A) Cu_2_O NNs and C/Cu_2_O NNs; (B) TiO_2−*x*_ NRs. Raman spectra of (C) C/Cu_2_O NNs; (D) TiO_2−*x*_ NRs.

Cu_2_O NNs and TiO_2−*x*_ NRs were characterized by XRD and Raman spectroscopy to confirm the structure and nanocrystalline nature. Although XRD pattern of the synthesized C/Cu_2_O NNs with 10 mg mL^−1^ dextrose showed a small shift in 2θ angle compared to that of Cu_2_O NNs; they both showed elemental Cu peaks attributed to the Cu foil substrate and a single cubic Cu_2_O phase (ICDD#00-05-0667) with a preferential orientation along the (111) orientation (Fig. 2A).

A slight shift towards lower 2θ angle could be due to residual stress due to the presence of carbon. The XRD results of TiO_2−*x*_ NRs (Fig. 2B) indicated a mixture of oxygen-deficient titanium oxide (TiO_0.5_) (ICDD#01-073-1570) and TiO_2_ anatase phase (ICSD#98-016-8140). These results suggest that the synthesized TiO_2−*x*_ NRs (*x* = 0, 1.5) consist of both amorphous and crystalline forms of rutile and anatase phases. Raman frequencies of C/Cu_2_O NNs (Fig. 2C) however only show peaks of Cu_2_O,^45^ probably due to the low contribution of intensity from the carbon layer. Additional Raman spectra of Cu foil, Cu(OH)_2_ NNs, Cu_2_O NNs, and C/Cu_2_O NNs prepared with other dextrose concentrations are shown in Fig. S1 (ESI).† TiO_2−*x*_ NRs' Raman spectra (Fig. 2D) showed a rutile phase with prominent peaks at 441 cm^−1^ (E_g_), 608 cm^−1^ (A_1_g), a weak peak at 140 cm^−1^ (B_1_g) and 236 cm^−1^ from a second-order effect (SOE).^46,47^ Thus, from Fig. 2D, it indicates that TiO_2−*x*_ first forms as amorphous NRs and then crystalline NRs grow on top of them. Although anatase TiO_2_ is usually more photoactive than rutile TiO_2_, previous studies have shown that the rutile phase shows a lower charge recombination rate.^48^ Recent studies have also revealed an increase in photoactivity in TiO_2_ samples that exhibit a mixture of anatase and rutile TiO_2_ phases due to electron transfer from the rutile states to the anatase states which are lower in energy or *vice versa*.^49^

**Fig. 3:**
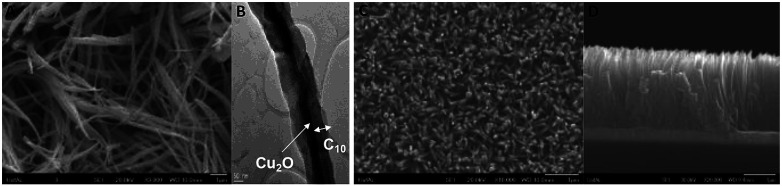
(A) SEM image of C/Cu_2_O NNs; (B) TEM image of a C/Cu_2_O NN; (C) top and (D) cross-sectional SEM images of 20 h TiO_2−*x*_ NRs on FTO.

Fig. 3A and B show the respective SEM and TEM images of C/Cu_2_O NNs synthesized using 10 mg mL^−1^ dextrose. C/Cu_2_O NNs are ∼5 μm in length and 400 nm in diameter. Fig. 3C and D reveal uniform hydrothermal growth of TiO_2−*x*_ NRs grown on FTO for 20 h with average NR thickness of ∼2 μm. C/Cu_2_O NNs were synthesized by anodization of Cu foil from KOH solution at a constant current of 10 mA to obtain uniformly distributed Cu(OH)_2_ NNs according to eqn (2). The SEM images of Cu(OH)_2_ NNs prepared at different anodization durations, and their corresponding photocurrent responses are shown in Fig. S2 and S3A,† respectively. The formation of carbon modified-Cu_2_O NNs was achieved by the dehydration and oxygen removal of Cu(OH)_2_ NNs upon annealing in the presence of dextrose and nitrogen (eqn (3)). During this process, dextrose is believed to dehydrate, cross-link, aromatize, and then carbonize to form a thin protective layer (Fig. 3B) that covers and protects the NNs morphology.^28^

2 Cu + 2OH^−^ → Cu(OH)_2_ + 2e^−^

3
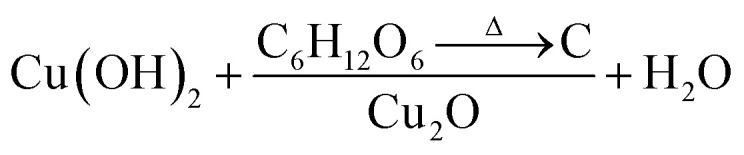


In the absence of the dextrose solution, the resulting Cu_2_O nanoneedles showed curly-like morphology but with similar dimensions (Fig. S4, ESI†). Synthesized at different durations (6–20 h), TiO_2−*x*_ NRs first form a TiO_2_ thick film having NR arrays that were approximately 5–7 μm in length and 200–400 nm in diameter. The NR length increased with the hydrothermal duration (Fig. S4†). Since Cu_2_O is known to suffer from photo-corrosion at the Cu_2_O/aqueous solution interface; a thin protective carbon layer helps increase conductivity and stability, thereby enhancing the charge transfer and separation at the solid–liquid interface for water reduction.^28^ A series of carbon-modified Cu_2_O NNs were prepared by soaking Cu(OH)_2_ NNs in different dextrose concentrations (1–50 mg mL^−1^) overnight. Although all C/Cu_2_O NNs showed better photocatalytic activity compared to unmodified Cu_2_O NNs, C/Cu_2_O NNs synthesized with 10 mg mL^−1^ dextrose showed optimum activity (Fig. S3B†). Cu_2_O samples were further characterized using cyclic voltammetry. As shown in Fig. S5,† a reduction peak of Cu^2+^ to Cu^+^ is observed around −0.20 V *vs.* Ag/AgCl for both Cu foil (Fig. S5A†) and Cu(OH)_2_ NNs (Fig. S5B†) samples. The reduction peak is shifted towards a more negative potential (−0.50 V *vs.* Ag/AgCl) for the unmodified Cu_2_O NNs (Fig. S5C†), and the shift can be attributed to the increase in charge diffusion polarization compared to Cu(OH)_2_ NNs and Cu foil samples.^50^ The absence of reduction peak for the carbon-modified Cu_2_O NNs (Fig. S4D†) confirms the role of the carbon layer in protecting the nanoneedle morphology. The photocurrent responses of different TiO_2−*x*_ NRs (6–20 h) were recorded in a three-electrode system under the back-side (FTO side) and the front-side (NRs side) illuminations (Fig. S6†). Although the average back-side illumination response is almost half the front-side response, TiO_2−*x*_ NRs grown for 12 and 20 h were further investigated for effect of the NR length on the overall efficiency of the tandem PEC cell. The reason for varied photocurrent responses for different TiO_2−*x*_ NRs under different illumination conditions is explained on the basis of few factors that includes light penetration depth (optical thickness), type of illumination (front or back), and hole diffusion length. According to Beer–Lambert's law, the film thickness must be optimized for maximum electrode performance. The absorbed light flux depends on the absorption length of the light impinging onto the photoelectrode. In other words, the optical thickness decides the ability of the photons to penetrate inside the film. Under front-side illumination, while traversing through the NRs, most photons are absorbed near the surface and are possibly used up in the water oxidation reaction. On the other hand, during backside illumination through FTO substrate, the photogenerated holes have to travel longer distance upon separation to reach the surface for water oxidation reaction, depending on the NR lengths. Under backside illumination, the sluggish water oxidation response from TiO_2_-12 h electrode at lower potentials compared to TiO_2_-15 h electrode is due to poor charge collection possibly due to the presence of defects trapping the holes and a poor interface between FTO and the base of TiO_2_ NRs. However, both TiO_2_-12 h and TiO_2_-15 h electrodes perform similarly at higher applied potentials. The reason for higher performance from TiO_2_-12 h electrode under front-side illumination could be due to efficient charge separation from optimal length of TiO_2_ NRs.

**Fig. 4:**
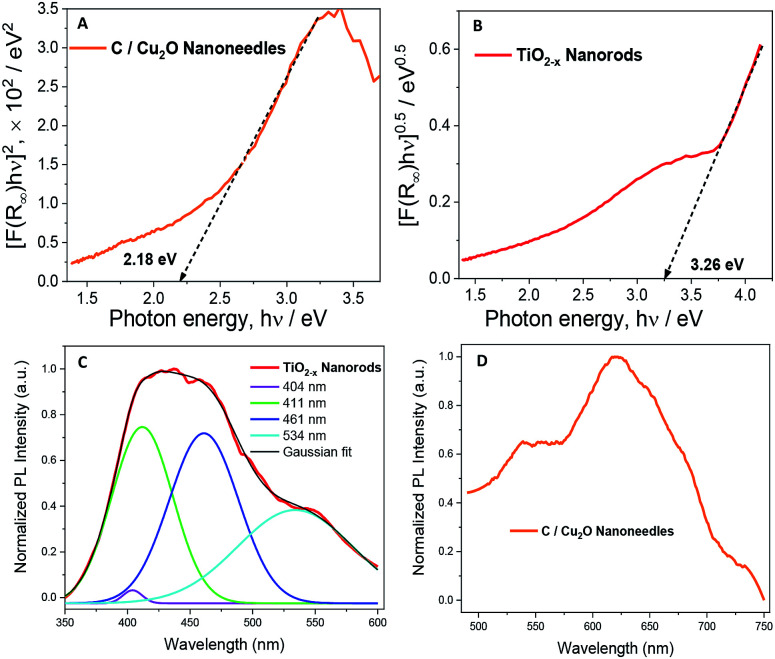
Tauc plots revealing band gap energies of (A) C/Cu_2_O NNs and (B) TiO_2−*x*_ NRs. Normalized emission spectra of (C) TiO_2−*x*_ NRs and (D) C/Cu_2_O NNs. The excitation wavelength of 365 nm and 470 nm for TiO_2−*x*_ NRs and C/Cu_2_O NNs, respectively. For emission measurements, the nanostructured films were scratched off, dissolved in ethanol, then drop-casted onto an indium tin oxide (ITO) glass substrate.

Fig. 4A and B show the Tauc plots of C/Cu_2_O NNs and TiO_2−*x*_ NRs, respectively. The DRS measurements were transformed using the Kubelka–Munk function (Fig. S7†), and a Tauc plot was used to estimate the band gap (*E*_g_) values of 2.18 eV for C/Cu_2_O NNs and 3.26 eV for TiO_2−*x*_ NRs.^28,51,52^ The photoelectrodes were further analysed by fluorescence spectroscopy to investigate the recombination processes of photogenerated carriers. TiO_2−*x*_ NRs, under excitation at 365 nm, showed a blue-green emission centered around 436 nm and a shoulder peak around 534 nm (Fig. 4C). The emission spectra were deconvoluted into four peaks: (1) 404 nm, attributed to the rutile structure of bulk TiO_2_ crystals; (2) 411 nm, attributed to the trapped excitons; (3) 461 nm and (4) 534 nm, which are due to oxygen vacancies at the surface of the NRs.^53–56^ The C/Cu_2_O NNs (Fig. 4D), excited at 470 nm, show a major exciton peak around 621 nm and a shoulder peak centered around 539 nm both usually attributed to phonon-assisted transitions in Cu_2_O samples confirming that no carbon elements were doped into the Cu_2_O lattice.^57^

**Fig. 5:**
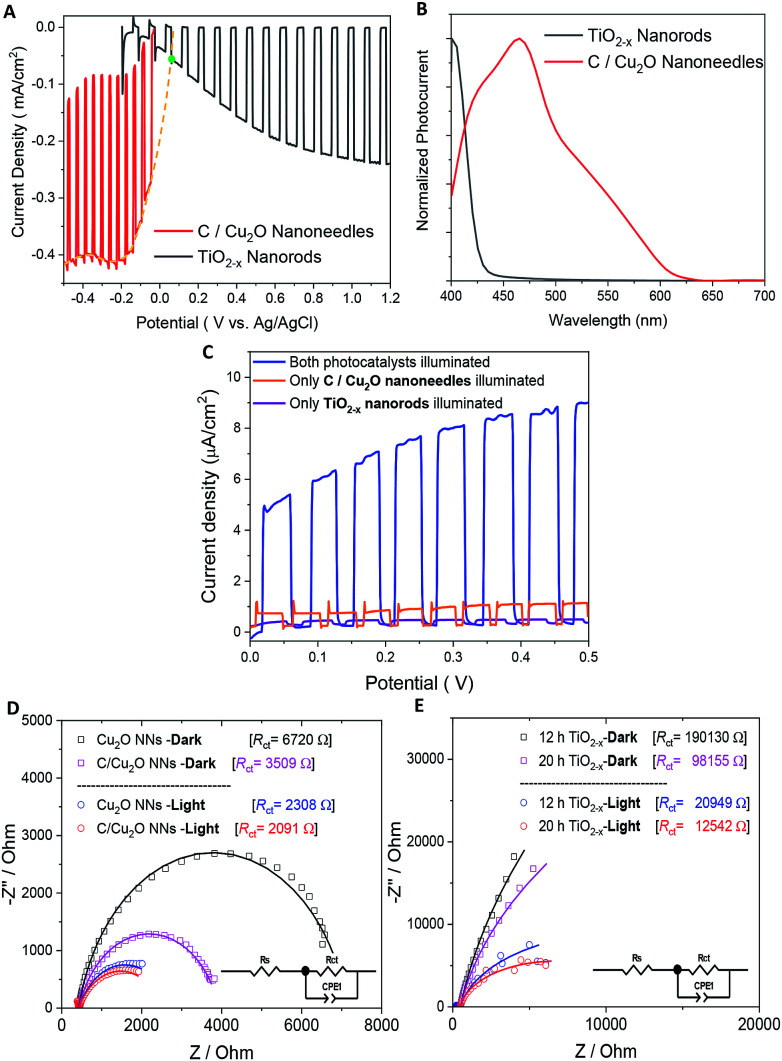
(A) Individual photocurrent responses of p-type C/Cu_2_O and n-type TiO_2−*x*_ (inverted) to determine the bias-free operating condition (a green dot depicts intersection point), (B) normalized spectral photocurrent responses of C/Cu_2_O NNs and TiO_2−*x*_ NRs, and (C) influence of individual illumination on the *J*–*V* curves of p-type C/Cu_2_O NNs‖n-type TiO_2−*x*_ NRs (20 h) tandem cells in parallel configuration. Nyquist plots of (D) Cu_2_O NNs with and without a protective carbon layer and (E) TiO_2−*x*_ NRs grown at 12 and 20 h in the dark and under the light. Electrolyte: N_2_-purged 0.5 M Na_2_SO_4_ in 0.1 M KH_2_PO_4_ (pH 5.0); light illumination: 1 sun (A.M 1.5, 100 mW cm^−2^).

Fig. 5A shows the individual photocurrent responses of p-type C/Cu_2_O NNs and n-type TiO_2−*x*_ NRs measured with three-electrode set-up. The photocurrent response of TiO_2_ NRs is inverted for convenience in order to identify the operating the current of Z-scheme solar water splitting system with a common intersection point, an approach analogous to typical load-line analyses of photovoltaic cells and resistive loads.^58^ The photocurrent turn-on potential of C/Cu_2_O NNs is more positive than that of n-type TiO_2−*x*_ NRs. The C/Cu_2_O NNs photocathode shows a net photocurrent density of ∼−300 μA cm^−2^ at 0 V *vs.* RHE (−0.5 V *vs.* Ag/AgCl). TiO_2_ shows a stable photocurrent density of 60 μA cm^−2^ at 1.23 V *vs.* RHE (0.8 V *vs.* Ag/AgCl). The cathodic photocurrents ranging from −240 μA cm^−2^ to −2.45 mA cm^−2^ at 0 V *vs.* RHE for anodized Cu_2_O NWs^23,24,27,31,59–62^ and anodic photocurrents ranging from 20 μA cm^−2^ to 0.5 mA cm^−2^ at 1.23 V *vs.* RHE for hydrothermally grown TiO_2_ NRs,^63–70^ have been reported in the literature. Nevertheless, our main goal in this paper is to demonstrate and establish unassisted Z-scheme solar water splitting in tandem configuration. Moreover, we envisage that still there remains much opportunity to optimize the individual performances of C/Cu_2_O and TiO_2−*x*_ photoelectrodes to improve the unassisted water splitting efficiency further. The intersection point in Fig. 5A shows the maximum operating current of 60 μA cm^−2^ for TiO_2−*x*_ NRs photoanode and C/Cu_2_O NNs photocathode. The action spectra of p-C/Cu_2_O NNs and n-TiO_2−*x*_ NRs (Fig. 5B) strongly suggests that the proposed tandem PEC cell can have broader sunlight absorption by extending the UV absorption response for the TiO_2−*x*_ NRs to visible absorption for the C/Cu_2_O NNs. The individual electrode contribution towards overall solar water splitting in a PEC cell was evaluated by measuring the *J*–*V* curves by exclusively illuminating one photoelectrode at a time and compared to the scenario when both electrodes were illuminated at simultaneously in a parallel configuration (Fig. 5C). It is clear that performances of both the photoelectrodes under individual illumination conditions while masking one another is minimal, even if they are added up. When both the electrodes are used and are illuminated simultaneously, there is a dramatic enhancement in the PEC performance. Hence, both photoelectrodes are needed to effectively produce and separate photogenerated holes and electrons that take part in the overall Z-scheme solar water splitting. EIS measurements for C/Cu_2_O NNs and TiO_2−*x*_ NRs photoelectrodes (Fig. 5D and E) were carried out in the dark and under simulated sunlight in a three-electrode configuration to obtain insights into the charge-transfer properties and the recombination processes of the photogenerated electron–hole pairs. The experimentally measured Nyquist plots were fitted using circuit elements consisting of one resistor and one RC circuit according to the standard Randles equivalent circuit (insets of Fig. 5D and E). Table S1 and S2† list the EIS parameters obtained from the fittings of Nyquist plots. The series resistance (*R*_s_) corresponding to the resistance of the electrolyte solution from working electrode to reference electrode is almost similar for all the samples (∼430 Ω for Cu_2_O samples and ∼495 Ω for TiO_2−*x*_ samples). The charge-transfer processes that dictate the photocurrent response of photoelectrodes is governed by the charge transfer resistance (*R*_ct_), which is lower for C/Cu_2_O NNs prepared with 10 mg mL^−1^ dextrose and TiO_2−*x*_ NRs (20 h) under both dark and light illumination conditions in comparison to unmodified Cu_2_O NNs and TiO_2−*x*_ NRs (12 h), respectively. Additionally, the corresponding double layer capacitance (*C*_dl_) is higher for both C/Cu_2_O NNs and TiO_2−*x*_ NRs (20 h). A constant phase shift element (CPE) is used to fit the equivalent circuit (insets of Fig. 5D and E) for capacitance *C*_dl_, meant for imperfect capacitance arising due to non-planar nature of the electrodes. Thus, carbon modification greatly improves the water splitting performance of Cu_2_O NNs due to efficient charge separation and the resistance of the TiO_2−*x*_ NRs decreases as the length increases.

**Fig. 6:**
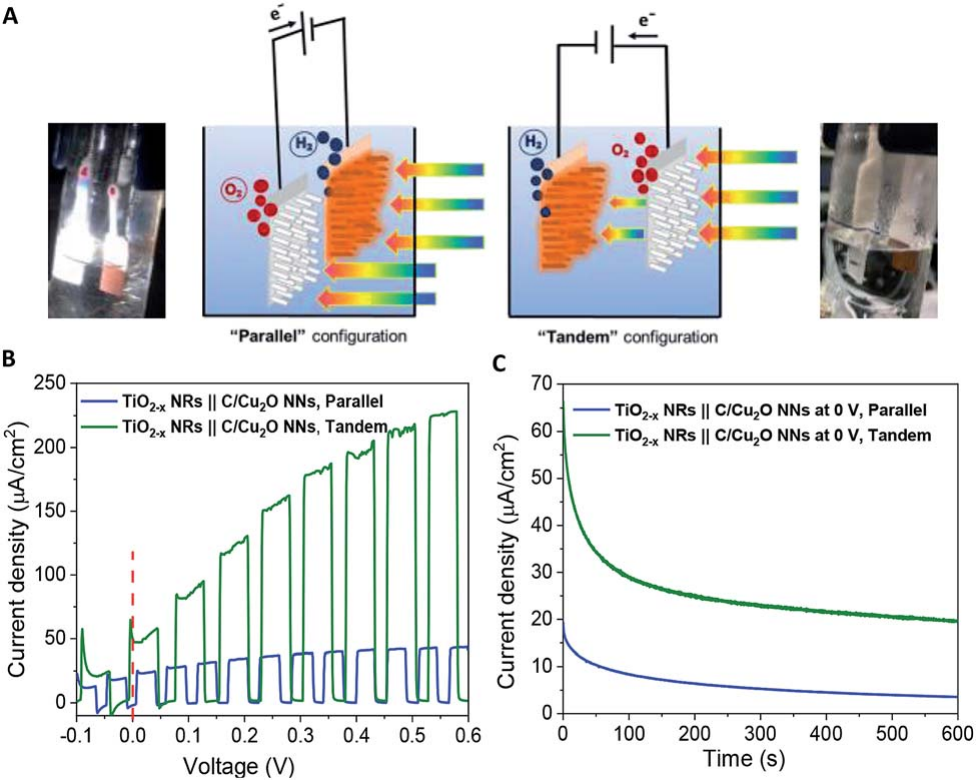
(A) Schematic illustration of the proposed p-type C/Cu_2_O NNs‖n-type TiO_2−*x*_ NRs tandem cell for Z-scheme water splitting. (B) *J*–*V* and (C) *J*–*t* curves of p-type C/Cu_2_O NNs and n-type TiO_2−*x*_ NRs (20 h) tandem cell at zero bias and without any sacrificial reagents. Electrolyte: N_2_-purged 0.5 M Na_2_SO_4_ in 0.1 M KH_2_PO_4_ electrolyte (pH 5.0); illumination: 1 sun (A.M 1.5, 100 mW cm^−2^).

**Fig. 7:**
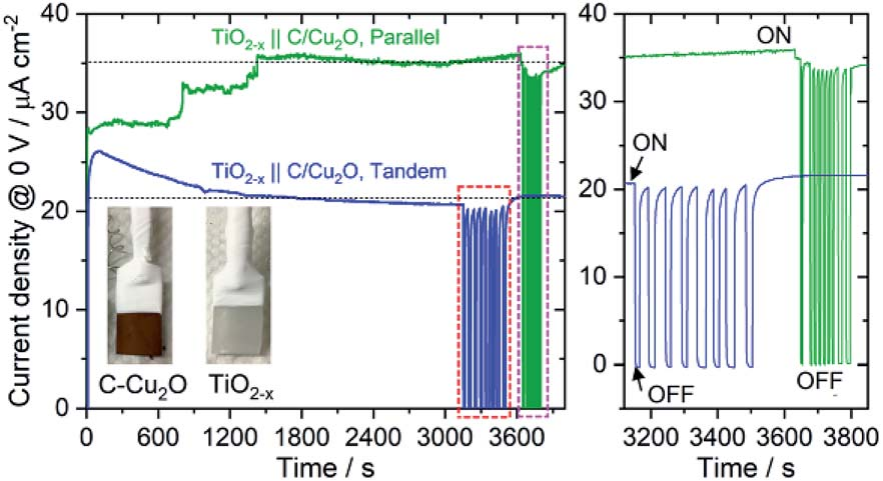
A long-term durability test of unassisted Z-scheme solar water splitting system involving n-type TiO_2−*x*_ NRs and p-type C/Cu_2_O NNs in tandem and parallel configurations, performed in N_2_-purged 0.5 M Na_2_SO_4_ phosphate-buffered electrolyte (pH 6.85) under 1 sun illumination. Light was chopped at the end to check the photoactivity response and photostability (right panel shows the magnified view of the chopped light response).

Fig. 6A shows the schematic setups of Z-scheme water splitting systems and the actual photographs of participating photoelectrodes in parallel and tandem configurations that were used to record the *J*–*V* curves of the two photoelectrodes in a non-sacrificial environment under a standard simulated solar light. Both configurations showed photocurrent responses indicating that the photoelectrons were efficiently shifted to the photocathode to produce the photocurrent.^51^ Additional *J*–*V* tests using TiO_2−*x*_ NRs of different thicknesses in both tandem and parallel configurations are shown in Fig. S8.† The cell photocurrent in the parallel configuration is relatively higher for 12 h TiO_2_ and drops for 20 h TiO_2_, but when compared at lower applied potentials, no discernible difference is seen for tandem configuration. The difference in tandem configuration is apparent only at higher applied potentials with relatively higher photocurrent for 20 h TiO_2_ contrary to the parallel configuration. Such anomaly can be explained based on charge collection and light penetration depths owing to different film thicknesses (TiO_2_ NR lengths). Longer the NR length, slower the charge collection rate. In parallel configuration and with the necessity to traverse smaller optical distance (for 12 h TiO_2_), the charge carriers are readily separated and contributes to the photocurrent without much losses than that for 20 h TiO_2_. On the contrary, in tandem configuration, the significant contribution in tandem configuration comes from thicker 20 h TiO_2_ than that of 12 h TiO_2_ and the photocurrent generation from C/Cu_2_O is limited by the number of charge carriers available for C/Cu_2_O upon their separation by TiO_2_. Under simulated solar light illumination, both C/Cu_2_O NNs and TiO_2−*x*_ NRs of the tandem PEC cell absorb photons to produce electrons and holes. The photogenerated electrons in the CB of the TiO_2−*x*_ NRs are transferred to the VB of the C/Cu_2_O NNs to recombine with the holes in C/Cu_2_O NNs. The holes in TiO_2−*x*_ NRs and the electrons in C/Cu_2_O NNs, on the other hand, migrate to the photoelectrode–electrolyte interface to participate in the overall Z-scheme water splitting.^13,52,71,72^ The photocurrent response of Z-scheme system involving C/Cu_2_O NNs and TiO_2−*x*_ NRs recorded with two-electrode set-up, shows relatively higher photoactivity for tandem configuration, especially at higher applied potentials (Fig. 6B). The photoelectrodes in tandem configuration showed better photoactivity compared to the parallel configuration, possibly due to the increased surface to volume ratio of the device. Fig. 6C shows the current response of unassisted water splitting cell involving p-type C/Cu_2_O NNs‖n-type TiO_2−*x*_ NRs in tandem and parallel configurations (using 20 h TiO_2−*x*_ NRs) performed in N_2_-purged 0.5 M Na_2_SO_4_ and 0.1 M KH_2_PO_4_ electrolyte (pH 5.0) under simulated 1 sun illumination with no external bias and any sacrificial reagents for 10 min. The photocurrent dropped to one-third of its initial photocurrent before reaching an average plateau of 20 μA cm^−2^ for the tandem configuration whereas the photocurrent for parallel configuration kept on decreasing over time indicating lower stability. Only a slight difference is seen for parallel and tandem configurations for unassisted tandem cell water splitting cell comprising 12 h TiO_2−*x*_ NR electrode (Fig. S9†), although the response is relatively stable in tandem configuration. In order to see the durability of unassisted Z-scheme solar water splitting system, a long-term photostability test was performed in both tandem and parallel configurations (Fig. 7). It was found that the electrodes are quite stable with no signs of any degradation over time. Although, tandem configuration response is better, the photoresponse of parallel configuration improved over time. The possible reason for this could be the release of loose C-layer on Cu_2_O NNs, which gets optimized over time. Chopped light response after an hour-long stability test, still shows the sustained photoactivity.

**Fig. 8:**
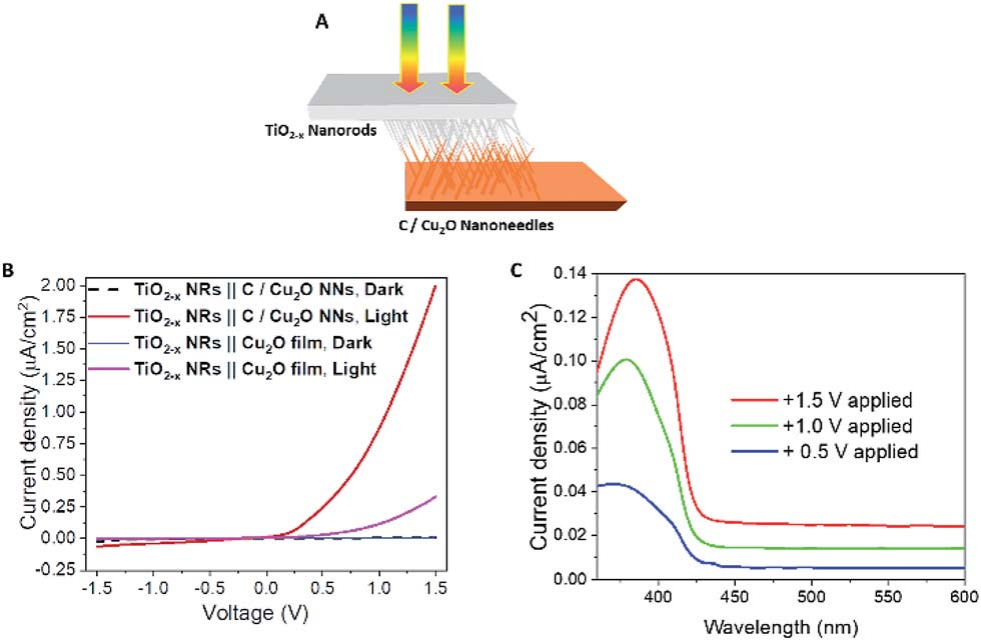
(A) Schematic illustration and (B) *J*–*V* curves of p-type C/Cu_2_O NNs‖n-type TiO_2−*x*_ NRs (20 h) heterojunction photodiode under 1 sun illumination (AM 1.5, 100 mW cm^−2^). (C) The spectral photocurrent response of p-type C/Cu_2_O NNs‖n-type TiO_2−*x*_ NRs (20 h) heterojunction measured at different applied potentials in a two-electrode system with TiO_2−*x*_ NRs biased positively for pronounced photocurrent response.

To further confirm the charge transfer and performance of the proposed tandem PEC cell, a flip-chip method (Fig. 8A)^73^ was used in the absence of any sacrificial redox species such as water. A light-activated heterojunction diode between C/Cu_2_O NNs and TiO_2−*x*_ NRs can be established with a low turn-on voltage around 0.15 V when these nanostructured electrodes are in intimate contact. Under forward bias, the photocurrent density increases nonlinearly up to ∼2 μA cm^−2^ and reaches almost six times more than the photoresponse from a planer Cu_2_O thin film because of its larger interfacial area in the nanostructure heterojunction (Fig. 8B). A leakage current close to 62 nA cm^−2^ was observed in the reversed bias at −1.5 V. *J*–*V* characteristics of the fabricated p–n junction diode showed a photo-dependent rectifying behavior with high sensitivity towards the UV light and a small photoresponse in the visible region from 430 to 600 nm (Fig. 8C). Such behavior is because of the higher population of carriers generated from TiO_2_ NRs than the bottom Cu_2_O NNs in the present light illumination configuration as shown in Fig. 8A.

In order to understand the charge transport and separation processes between C/Cu_2_O NNs and TiO_2−*x*_ NRs and possible charge transfer mechanism of the photogenerated charge carriers, the respective CB or VB edge positions and carrier concentrations of C/Cu_2_O and TiO_2−*x*_ are estimated from Mott–Schottky analysis using following relations.^74,75^4
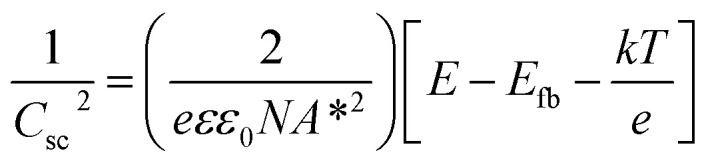
5
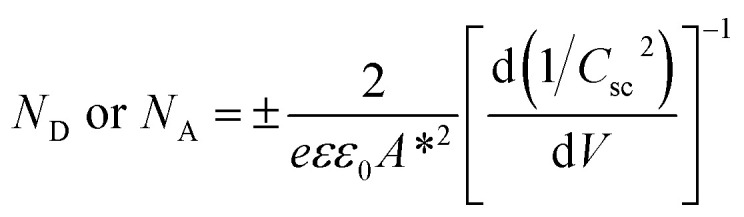
where *C*_sc_ is the space charge capacitance, *e* is the electron charge, *ε* is the dielectric constant of the semiconductor (*ε* = 170 for TiO_2_ (ref. 76) and *ε* = 7.6 for Cu_2_O (ref. 26)), *ε*_0_ is the permittivity of the free space, *N* is the donor or acceptor density (*N*_D_ or *N*_A_), *E* is the applied potential, *E*_fb_ is the flat-band potential that equals the Fermi energy when semiconductor band bending vanishes at flat-band conditions,^77,78^A* is the area of the electrode in contact, *k* is the Boltzmann constant, and *T* is the absolute temperature. When the linear region of the Mott–Schottky plot is extrapolated to *X*-axis at 1/*C*_sc_^2^ = 0, the intercept at the *X*-axis leads to the quantity (*E*_fb_ + *kT*/*e*), from which the flat-band potential values are determined (Fig. S10†). The *E*_fb_ values estimated from MS plot for TiO_2−*x*_ NR and C/Cu_2_O NN electrodes are 0.13 and 0.54 V *vs.* RHE, respectively, which are in agreement with the literature values for TiO_2_ (ref. 75, 76 and 79) and Cu_2_O.^80,81^ The positive and negative slopes in the MS plots or +ve and −ve sign in eqn (2) confirm the n-type and p-type conductivity of TiO_2−*x*_ and C/Cu_2_O electrodes, respectively. The corresponding *N*_D_ and *N*_A_ carrier concentrations estimated for TiO_2−*x*_ and C/Cu_2_O are 2.103 × 10^17^ cm^−3^ and 2.262 × 10^20^ cm^−3^, respectively, which are also in line with the reported values for TiO_2_ (ref. 75 and 76) and Cu_2_O.^82–84^ A relatively high value of *N*_A_, which is otherwise on the order of 10^15^ to 10^17^ cm^−3^,^85^ has been attributed to the higher density of vacancies, the hydrous nature of oxide nanomaterials, or use of conducting substrates.^82–84^

**Fig. 9:**
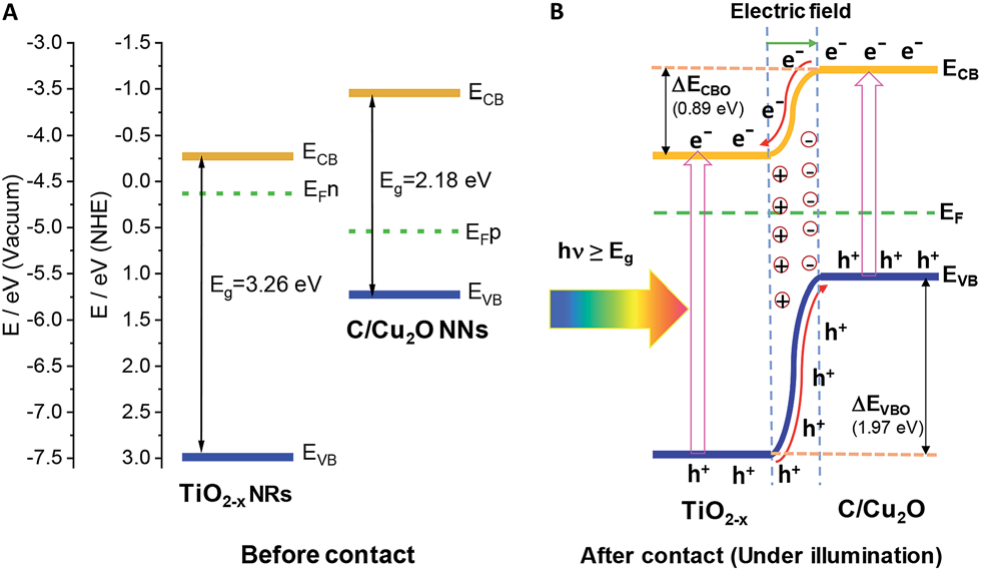
Energy band diagrams of p-type C/Cu_2_O NNs and n-type TiO_2−*x*_ NRs (20 h) (A) before and (B) after intimate contact to form a p–n heterojunction.

The Fermi energy levels, CB or VB band positions are estimated based on MS analysis and optical absorption studies and the energy band diagrams are constructed. Fig. 9A and B show the reconstructed energy band diagram before contact and after contact under illumination with a type-II band alignment. After contact, the Fermi levels of C/Cu_2_O NNs and TiO_2−*x*_ NRs moves up and down until an equilibrium is established. As a result of the formation of a p–n heterojunction, an electric field is established at the interface. A possible charge transfer mechanism in the present electrode configuration is hypothesized as follows: (1) upon solar irradiation at the side of TiO_2−*x*_ NRs, UV part of light is generally absorbed to generate electron–hole pairs mostly on the side of TiO_2−*x*_ NRs electrode and partially transmitted visible light is absorbed by the underlying C/Cu_2_O NNs; (2) the photogenerated electrons in both C/Cu_2_O NNs and TiO_2−*x*_ NRs are separated from the holes followed by collection at the top of CB on the TiO_2−*x*_ NRs side, with holes collected at the bottom VB of C/Cu_2_O NNs. This p–n heterojunction is similar to the forward bias observed in p–n diodes as the electrons only need to overcome a small energy barrier, *i.e.* CB offset (Δ*E*_CBO_) of 0.89 eV; whereas, holes are limited by a higher energy barrier VB offset (Δ*E*_VBO_) = 1.97 eV in the reverse bias exhibiting a low current response. Aguirre *et al.* recently obtained similar Δ*E*_CBO_ and Δ*E*_VBO_ values of 0.81 and 1.91 eV, respectively, for TiO_2_–Cu_2_O heterojunction system.^86^ Although the p–n heterojunction photodiode does not suffer from sluggish redox reactions and the corrosion effects in water encountered in the proposed PEC tandem cell; further optimization to maximize contact between the two photoelectrodes could increase the charge separation, stability and ultimately the photocurrent response from the p–n heterojunction photodiode. To improve the tandem cell efficiency further, the absorption of TiO_2_ NRs can be extended in the visible region by decreasing the band gap *via* hydrogen doping,^87^ or through surface plasmon resonance (SPR) effect of Au nanostructures.^88^ Alternatively, a Z-scheme photocathode can be formed by depositing Cu_2_O on Au-incorporated TiO_2_ NRs array to improve the overall charge separation, the carrier density, and the kinetics of electron injection into electrolyte for enhancing the photoactivity toward solar-to-fuel energy conversion.^16^ Another approach would be to combine TiO_2_ NRs with chalcogenide to improve the aligned hole transport and charge-transfer kinetics.^89^

## 4. Conclusions

In this work, a self-biased, UV-vis light-responsive tandem cell for Z-scheme solar water splitting was established using two photoelectrodes, *viz.* a p-type carbon-modified Cu_2_O NNs and n-type TiO_2−*x*_ NRs. The carbon layer was proven to protect the Cu_2_O NN morphology as well as improve the charge separation by imparting additional conductivity, while the presence of oxygen vacancies facilitated the charge transfer in TiO_2−*x*_ NRs. Such tandem cell exhibited unassisted solar driven Z-scheme water splitting with a photocurrent activity of 64.7 μA cm^−2^ that gradually decreased over time. Although the tandem cell performance did not use any sacrificial reagents or redox mediators, the overall tandem performance is still limited by the C/Cu_2_O NNs performance and poor visible-light response from TiO_2_. Further improvements to the system are still necessary to increase the overall performance. Simultaneously, the nanostructured heterojunction diode made of C/Cu_2_O NNs and TiO_2−*x*_ NRs demonstrated a current density of 2 μA cm^−2^ at 1.5 V with a 62 nA cm^−2^ leakage current at −1.5 V. These findings could open new pathways to develop low-cost and efficient unassisted solar water splitting systems. Photodiode characteristics of the nanostructured p–n junction electrode is not only promising towards PEC studies for solar water splitting but also would potentially benefit other electronic devices such as ultrasensitive molecular sensing and optoelectronics.

## Conflicts of interest

There are no conflicts to declare.

## Supplementary Material

RA-009-C8RA09403A-s001
