# Correction: Photoelectrochemical study of carbon-modified p-type Cu_2_O nanoneedles and n-type TiO_2−*x*_ nanorods for Z-scheme solar water splitting in a tandem cell configuration

**DOI:** 10.1039/c9ra90035g

**Published:** 2019-05-17

**Authors:** Nelly Kaneza, Pravin S. Shinde, Yanxiao Ma, Shanlin Pan

**Affiliations:** Department of Chemistry and Biochemistry, The University of Alabama Tuscaloosa AL 35487-0336 USA span1@ua.edu

## Abstract

Correction for ‘Photoelectrochemical study of carbon-modified p-type Cu_2_O nanoneedles and n-type TiO_2−*x*_ nanorods for Z-scheme solar water splitting in a tandem cell configuration’ by Nelly Kaneza *et al.*, *RSC Adv.*, 2019, **9**, 13576–13585.

The authors regret that eqn (3) is shown incorrectly in the original article. The correct equation is shown here.3
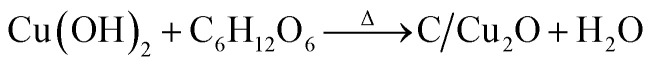


The Royal Society of Chemistry apologises for these errors and any consequent inconvenience to authors and readers.

## Supplementary Material

