# Supplementary material for: Photoelectrochemical study of carbon-modified p-type Cu2O nanoneedles and n-type TiO2−x nanorods for Z-scheme solar water splitting in a tandem cell configuration
Source: RSC Adv. 2019 May 2;9(24):13576–85. doi: 10.1039/c8ra09403a (PMC9063928; doi:10.1039/c8ra09403a)
Supplement: RA-009-C8RA09403A-s001 [file RA-009-C8RA09403A-s001.pdf]

Electronic Supplementary Material (ESI) for RSC Advances.
This journal is © The Royal Society of Chemistry 2019

Supporting Information

Photoelectrochemical Study of Carbon-Modified *p*-type Cu_2O Nanoneedles and *n*-type TiO_{2-x} Nanorods for Z-scheme Solar Water Splitting in Tandem Cell Configuration

Nelly Kaneza, Pravin S. Shinde, Yanxiao Ma, Shanlin Pan*

Department of Chemistry and Biochemisrty, The University of Alabama, Tuscaloosa, Alabama 35487

*Corresponding Author: span1@ua.edu, 1-205-348-6381

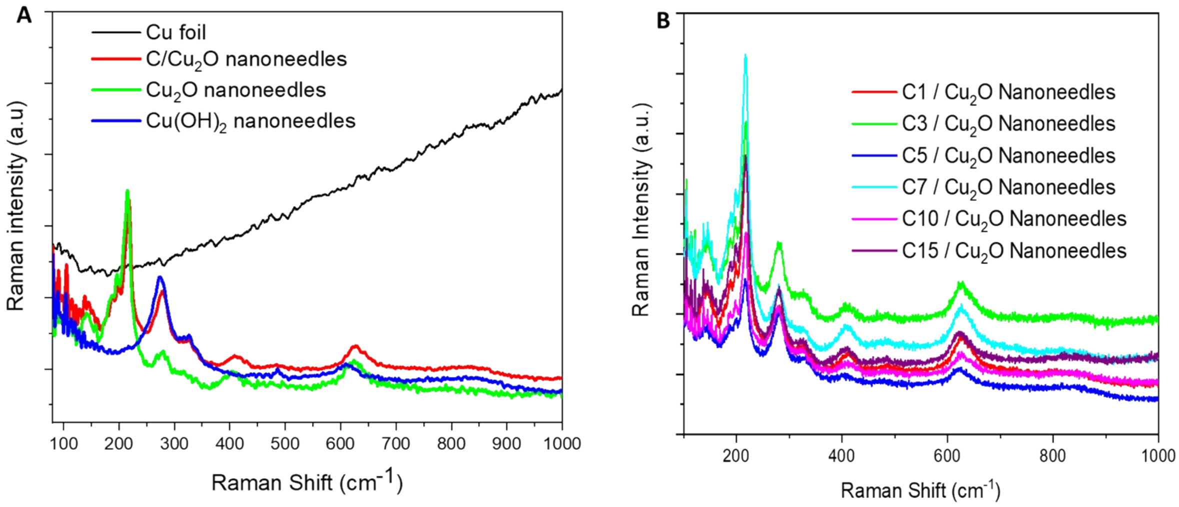

Fig. S1: (**A**) Raman spectra of Cu foil, $Cu(OH)_2$ NNs, Cu_2O NNs and C/Cu_2O NNs. (**B**) Raman spectra of different carbon-modified (1-15 mg mL^{-1})/Cu_2O NNs. Laser excitation wavelength=532 nm.

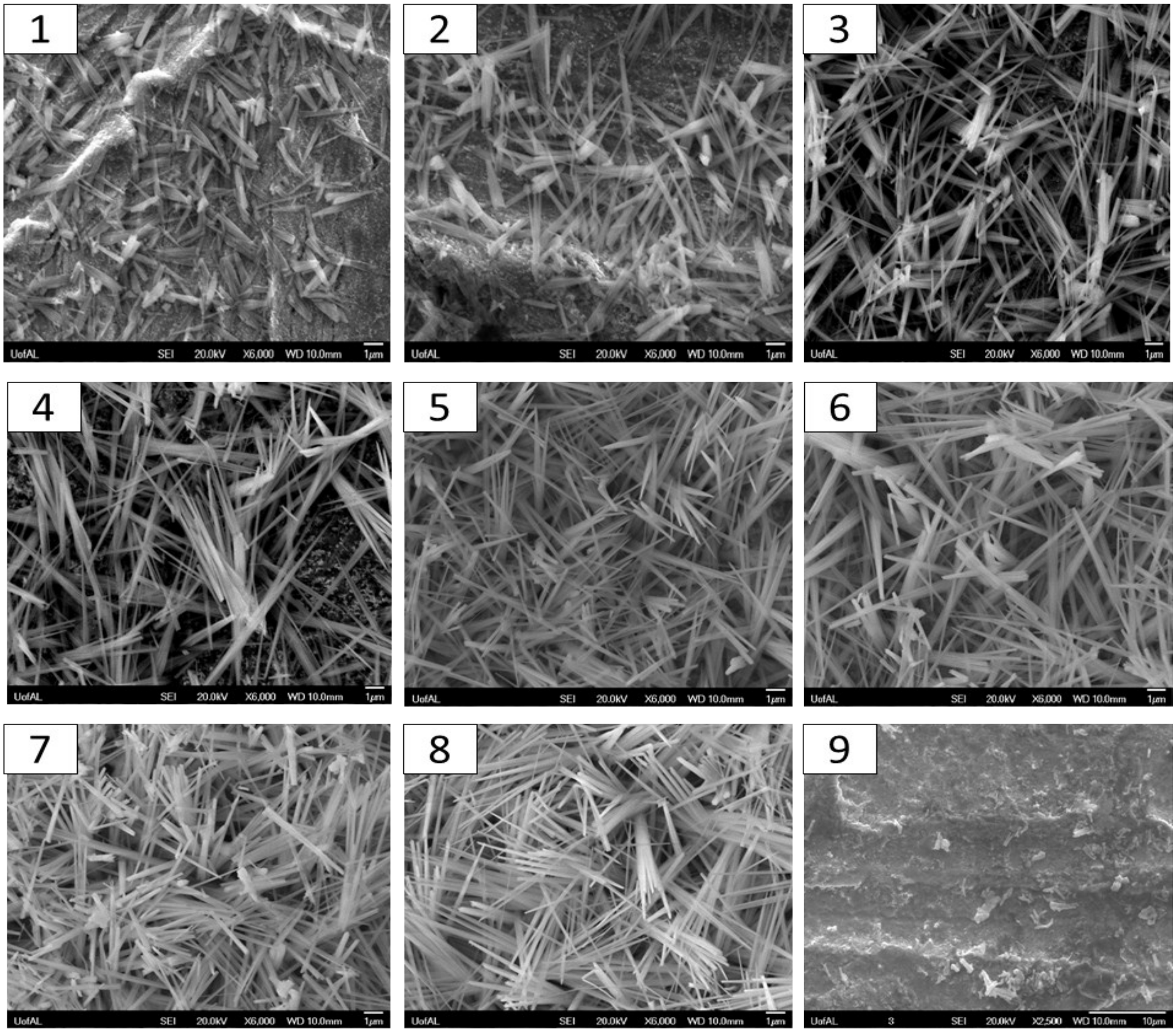

Fig. S2: SEM images of $Cu(OH)_2$ NNs synthesized at 2.0 V and 10 mA for different anodization durations (1-9 min) in 2 M KOH.

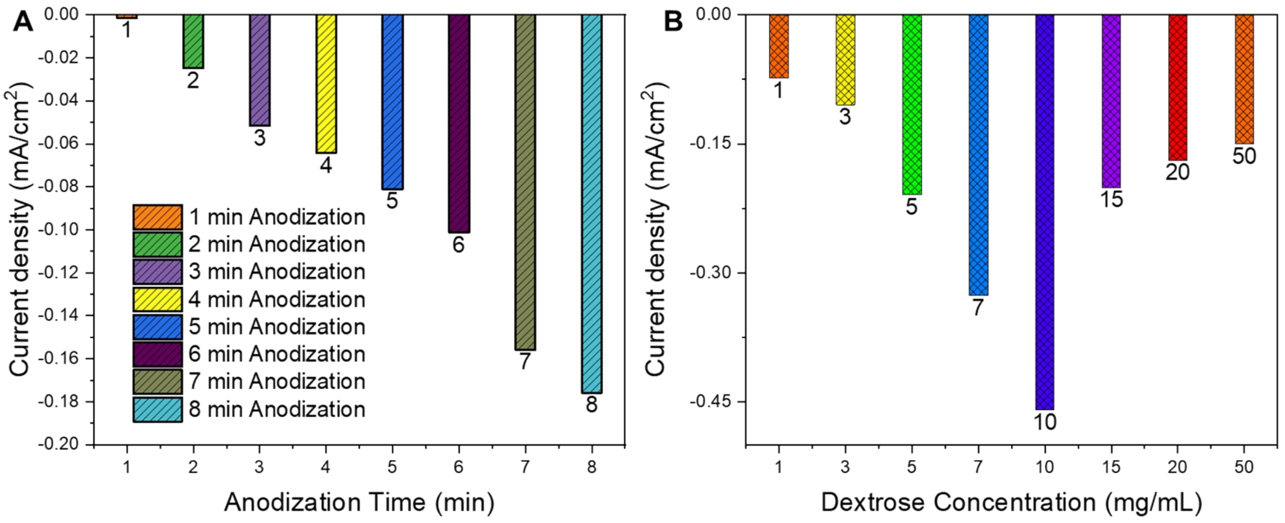

Fig. S3: (A) Photocurrent responses of C/Cu_2O NNs obtained from $Cu(OH)_2$ NNs anodized at different durations (1-8 min) and (**B**) Effect of dextrose concentration (1- 50 mg mL^{-1}) on formation of C_d/Cu_2O NNs from $Cu(OH)_2$ NNs anodized at 8 min. All current densities were recorded at -0.50 V *vs*. Ag/AgCl.

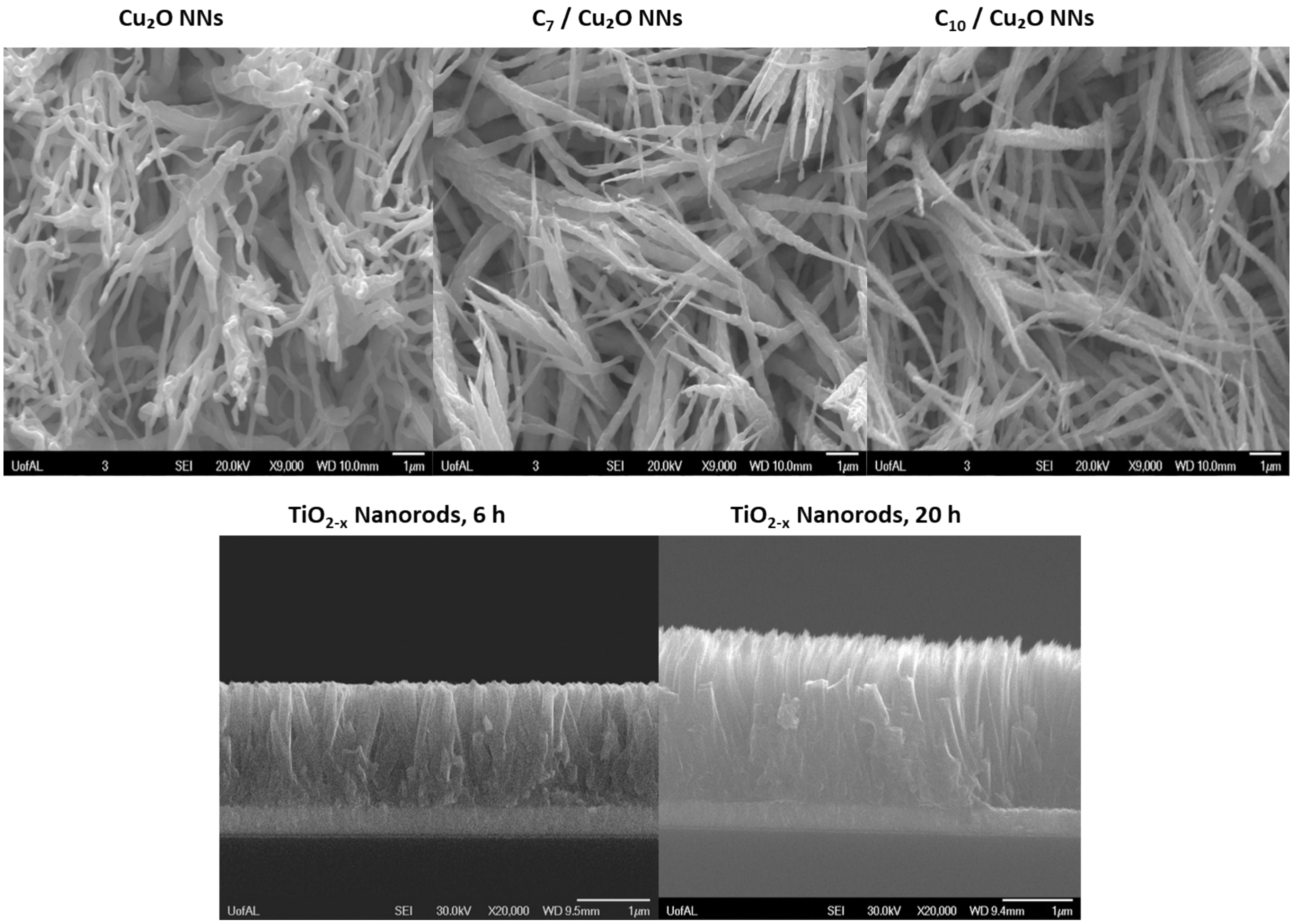

Fig. S4: SEM images of Cu_2O, C_7/Cu_2O, and C_{10}/Cu_2O NNs obtained from $Cu(OH)_2$ NNs anodized for 8 min; and cross-sectional SEM images of oxygen deficient $TiO_{2\text{-}x}$ NRs hydrothermally grown for 6 and 20 h.

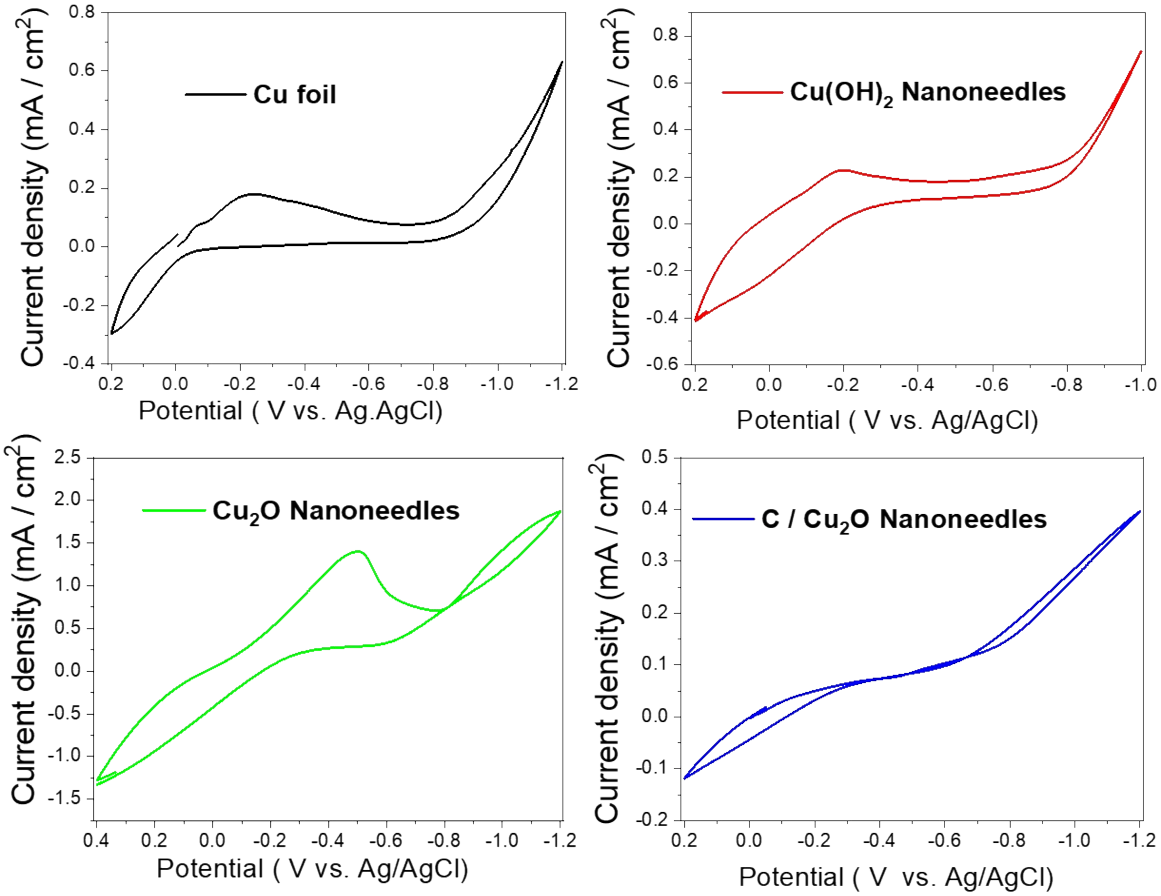

Fig. S5: Cyclic voltammetry of (**A**) Cu foil, (**B**) $Cu(OH)_2$ NNs, (**C**) Cu_2O NNs, and (**D**) C/Cu_2O NNs in 0.5 M Na_2SO_4 and 0.1 M KH_2PO_4 at a scan rate of 100 mV s^{-1}. Experimental data was measured in a three-electrode setup with Ag/AgCl as the reference electrode and graphite as the counter electrode.

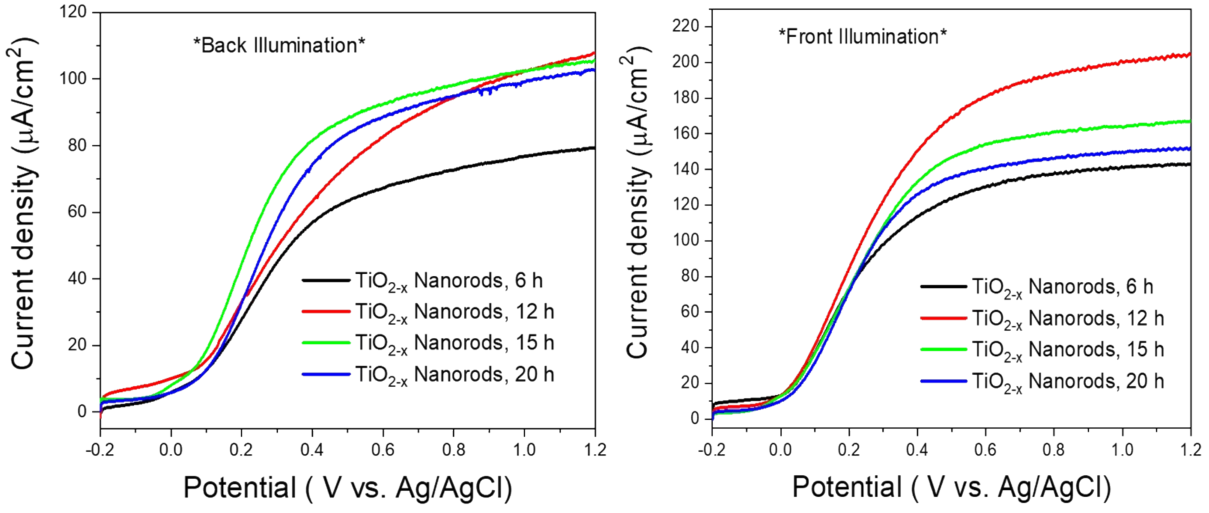

Fig. S6: *J-V* curves of oxygen-deficient TiO_{2-x} nanorods hydrothermally grown for different durations (6-20 h) in N_2-purged 0.5 M Na_2SO_4 and 0.1 M KH_2PO_4 (pH 5.0) under simulated 1 sun illumination (A.M 1.5). Front illumination: nanorods side; Back illumination: FTO side.

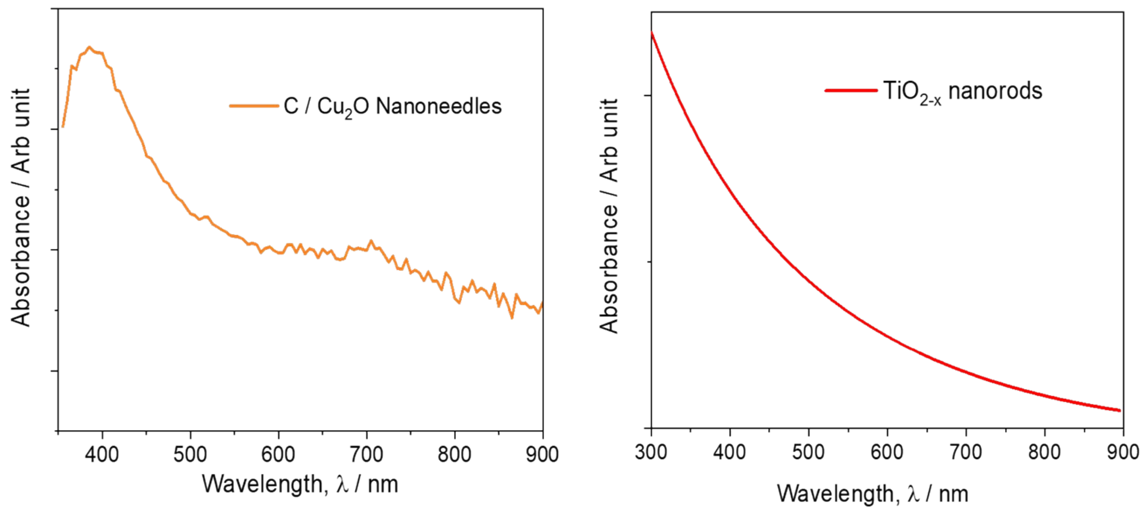

Fig. S7: UV-Vis diffuse reflectance spectra (DRS) of C/Cu_2O NNs and TiO_{2-x} NRs.

Table S1: Electrochemical impedance spectroscopy (EIS) parameters obtained for TiO_{2-x} NRs fabricated with hydrothermal growth times of 12 and 20 h.

**Sample**	R_s **(Ω)**	R_{ct} **(Ω)**	***CPE* or** C_{dl} **(μF)**
12 h TiO_{2-x} NRs Dark	496.5	1,90,130	2.259
12 h TiO_{2-x} NRs Light	495.2	20,949	6.366
20 h TiO_{2-x} NRs Dark	492.9	98,155	3.443
20 h TiO_{2-x} NRs Light	497.4	12,542	11.083

Table S2: Electrochemical impedance spectroscopy (EIS) parameters obtained for Cu_2O NNs with and without a protective carbon layer.

**Sample**	R_s **(Ω)**	R_{ct} **(Ω)**	***CPE* or** C_{dl} **(μF)**
Cu_2O NNs Dark	427.5	6720	8.118
Cu_2O NNs Light	430.7	2308	179.390
C/Cu_2O NNs Dark	429.5	3509	22.697
C/Cu_2O NNs Light	425.7	2091	230.150

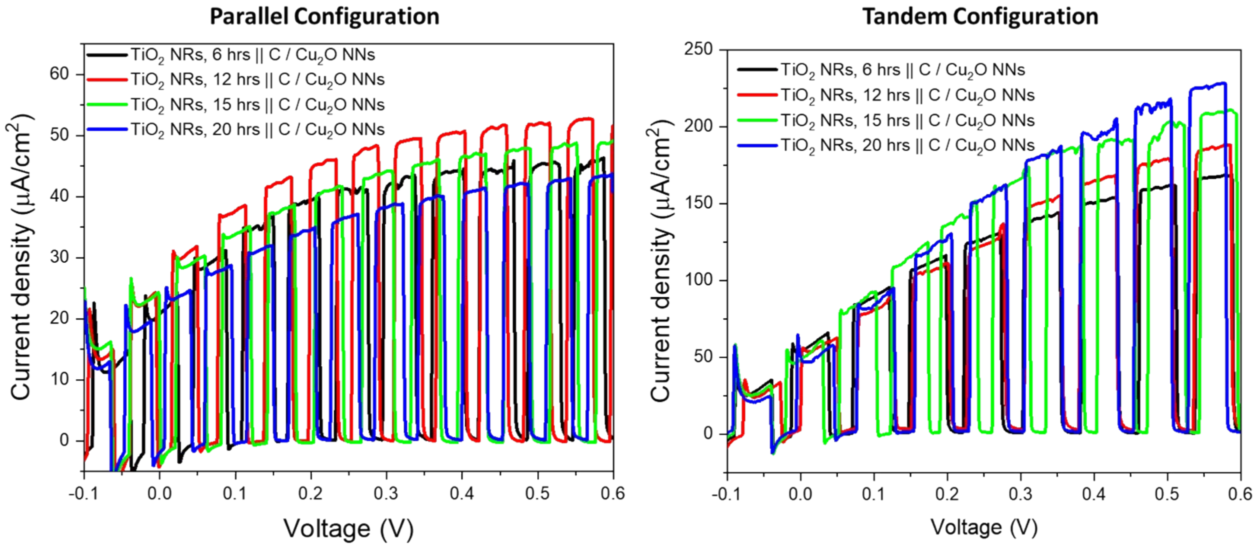

Fig. S8: *J-V* curves of *p*-type C/Cu_2O NNs/*n*-type TiO_{2-x} NRs (6-20 h) tandem cells in N_2-purged 0.5 M Na_2SO_4 and 0.1 M KH_2PO_4 electrolyte (pH 5.0) under simulated 1 sun illumination (A.M 1.5). The proposed tandem cells were placed in parallel or tandem configuration.

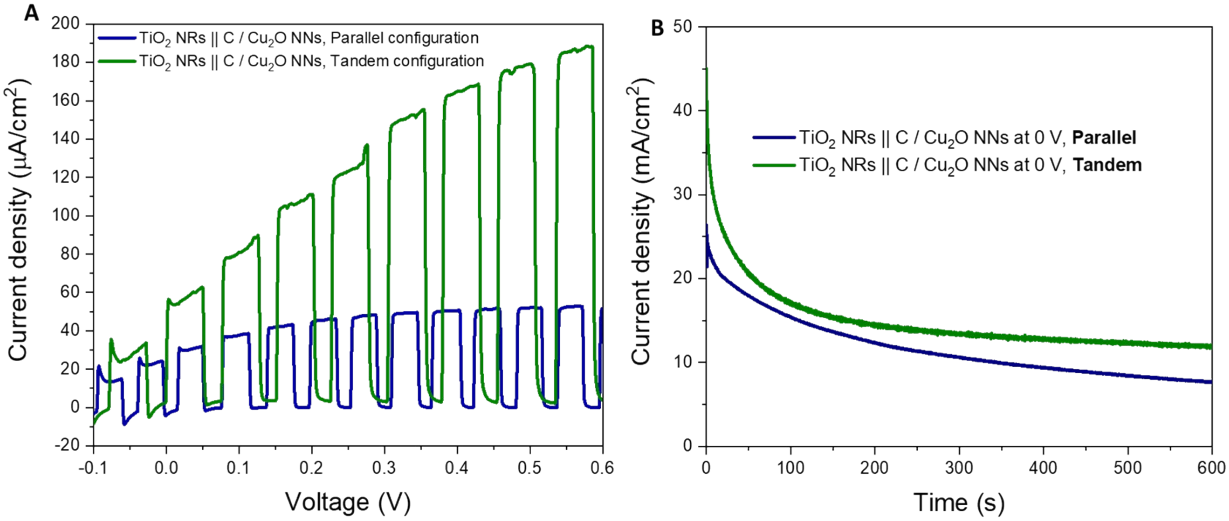

Fig. S9: (**A**) *J-V* and (**B**) *J-t* curves of *p*-type C/Cu_2O NNs/*n*-type TiO_{2-x} NRs (12 h) tandem cells in N_2-purged 0.5 M Na_2SO_4 and 0.1 M KH_2PO_4 electrolyte (pH 5.0) under simulated 1 sun illumination (A.M 1.5) at zero bias and without any sacrificial reagents.

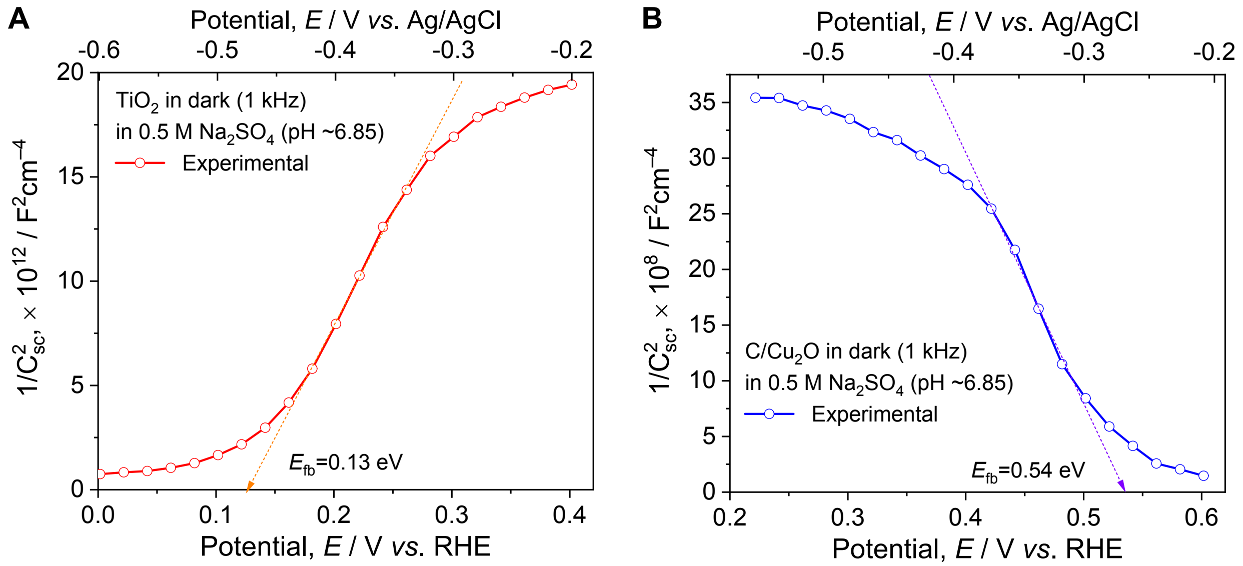

Fig. S10: Mott-Schottky plots of (**A**) *n*-type TiO2-x NRs and (**B**) *p*-type C/Cu_2O NNs measured from N_2-purged 0.5 M Na_2SO_4 phosphate-buffered electrolyte (pH 6.85) under dark condition at 1KHz, revealing the flat band potential (E_{fb}) values.
